# Topoisomerase I activity and sensitivity to camptothecin in breast cancer-derived cells: a comparative study

**DOI:** 10.1186/s12885-019-6371-0

**Published:** 2019-11-29

**Authors:** Cinzia Tesauro, Anne Katrine Simonsen, Marie Bech Andersen, Kamilla Wandsoe Petersen, Emil Laust Kristoffersen, Line Algreen, Noriko Yokoyama Hansen, Anne Bech Andersen, Ann Katrine Jakobsen, Magnus Stougaard, Pavel Gromov, Birgitta R. Knudsen, Irina Gromova

**Affiliations:** 1 Department of Molecular Biology and Genetics, Aarhus University, Aarhus, Denmark; 2 Present Address: Department of Biology, Copenhagen University, Copenhagen, Denmark; 3 Present Address: MRC Laboratory of Molecular Biology, Cambridge, UK; 4 Department of Pathology, Aarhus University Hospital, Aarhus, Denmark; 5 Genome Integrity Unit, Breast Cancer Biology Group, Danish Cancer Society Research Center, Copenhagen, Denmark

**Keywords:** Breast cancer, Breast cancer cell lines, Breast tumor subtypes, Topoisomerase I, Camptothecin sensivity

## Abstract

**Background:**

Camptothecin (CPT) and its derivatives are currently used as second- or third-line treatment for patients with endocrine-resistant breast cancer (BC). These drugs convert nuclear enzyme DNA topoisomerase I (TOP1) to a cell poison with the potential to damage DNA by increasing the half-life of TOP1-DNA cleavage complexes (TOP1cc), ultimately resulting in cell death. In small and non-randomized trials for BC, researchers have observed extensive variation in CPT response rates, ranging from 14 to 64%. This variability may be due to the absence of reliable selective parameters for patient stratification. BC cell lines may serve as feasible models for generation of functional criteria that may be used to predict drug sensitivity for patient stratification and, thus, lead to more appropriate applications of CPT in clinical trials. However, no study published to date has included a comparison of multiple relevant parameters and CPT response across cell lines corresponding to specific BC subtypes.

**Method:**

We evaluated the levels and possible associations of seven parameters including the status of the *TOP1* gene (i.e. amplification), TOP1 protein expression level, TOP1 activity and CPT susceptibility, activity of the tyrosyl-DNA phosphodiesterase 1 (TDP1), the cellular CPT response and the cellular growth rate across a representative panel of BC cell lines, which exemplifies three major BC subtypes: Luminal, HER2 and TNBC.

**Results:**

In all BC cell lines analyzed (without regard to subtype classification), we observed a significant overall correlation between growth rate and CPT response. In cell lines derived from Luminal and HER2 subtypes, we observed a correlation between *TOP1* gene copy number, TOP1 activity, and CPT response, although the data were too limited for statistical analyses. In cell lines representing Luminal and TNBC subtypes, we observed a direct correlation between TOP1 protein abundancy and levels of enzymatic activity. In all three subtypes (Luminal, HER2, and TNBC), TOP1 exhibits approximately the same susceptibility to CPT. Of the three subtypes examined, the TNBC-like cell lines exhibited the highest CPT sensitivity and were characterized by the fastest growth rate. This indicates that breast tumors belonging to the TNBC subtype, may benefit from treatment with CPT derivatives.

**Conclusion:**

TOP1 activity is not a marker for CPT sensitivity in breast cancer.

## Background

Breast cancer (BC) is the most frequent form of cancer among women [1]. The incidence of BC in Denmark is one of the highest in the world, with 4686 new cases annually [2]. Criteria for morphological BC subtyping have recently been refined and supplemented with complementary molecular classifications based on transcriptomic profiling and/or genomic analysis [3, 4]. The heterogeneity of BC, encompassing as many as 10 distinct intrinsic molecular subtypes with various morphological and molecular features, natural histories and responses to therapy, poses a significant challenge for the design of effective treatment regimens [5]. Currently, molecular targeted therapies are available only for estrogen receptor-positive (ER) and human epidermal growth factor receptor 2-positive (HER2) breast tumor [6–8]. However, a significant proportion (~ 30%) of primary tumors do not express ER or HER2. These tumors comprise the so-called triple-negative breast cancer (TNBC) group, which can currently be treated only with conventional chemotherapy [9–11]. TNBC tumors usually develop at a higher rate in young patients, tend to be high grade, and have a poor prognosis due to their generally aggressive nature and the lack of effective therapy [12, 13]. A small but significant number of patients with hormone-positive BC either do not respond or will acquire resistance to endocrine therapy during treatment and eventually develop disease recurrence [14]. Conventional chemotherapy remains the mainstay of treatment for such BC patients due to a lack of suitable molecular therapeutic targets. The combination of limited treatment options with the clinico-pathological heterogeneity of BC renders clinical management of such lesions extremely demanding [15]. Improved management of tumors that do not respond to any treatment is of vital importance [16].

Camptothecin (CPT) is the mother compound of an important class of drugs that specifically target the nuclear enzyme topoisomerase I (TOP1). The members of this class of compounds are currently among the most effective anti-cancer drugs for the treatment of advanced-stage gastrointestinal (colorectal and gastro-esophageal) malignancies, ovarian cancer, and recurrent small-cell lung cancer (SCL) [17–19]. Biologically, TOP1 plays essential roles in the maintenance of genomic integrity by releasing topological DNA stress that arises during transcription or replication. This is achieved with creation of a transient single-strand break in the DNA, which allows for the relaxation of DNA supercoiling [20]. Although the nicking-closing activity of TOP1 is remarkably fast (up to 6000 cycles per minute), the enzyme is susceptible to a number of drugs, including CPT, selectively when it is in covalent complex with DNA [21, 22].

CPT inhibits the re-ligation step of the TOP1 catalytic cycle, leading to the accumulation of cytotoxic covalent TOP1-DNA cleavage complexes (TOP1cc). The cytotoxic effect of TOP1cc is specific to the S-phase and is thought to reflect collision events between the replication machinery and TOP1cc, leading to DNA fragmentation and cell death [23]. Decreased expression of TOP1 protein poses a limitation to the number of TOP1cc that can be trapped. Reduced enzyme activity is associated with decreased generation of TOP1cc. The efficiency by which TOP1-mediated re-ligation is inhibited by CPT (i.e., the CPT susceptibility of TOP1) may also affect the number of accumulated TOP1cc and thereby modulate the cytotoxic effect of CPT. Enzyme activity and susceptibility to CPT may be regulated by means other than TOP1 transcription and translation [24, 25], such as post-translational modifications or interaction with other proteins [26–28]. Hence, for prediction of the cellular CPT response, it may be informative to measure expression level, activity and CPT susceptibility of the TOP1 enzyme as previously demonstrated in experiments with colorectal cancer cell lines [26] . Also, DNA repair proteins such as tyrosyl-DNA phosphodiesterase 1 (TDP1) that removes the protein moiety from trapped TOP1cc and facilitate sealing of the DNA nick by ligases before any permanent damage can be generated may affect the cellular CPT response [29, 30].

Consistent with the mechanism of CPT inhibition, high expression levels of TOP1 are associated with high CPT sensitivity [31, 32]. Conversely, decreased TOP1 expression has been linked to resistance to CPT [33, 34]. Recent research suggests that *TOP1* gene copy number may be used as an alternative to TOP1 protein expression as a predictive biomarker for stratification of patients with CPT-responsive colorectal cancer [35–37]. However, the results obtained from studies investigating such a possibility are inconsistent. The data published to date on the predictive validity of TOP1 protein expression in the adjuvant setting have been inconclusive [38].

The efficacy of CPT derivatives for the treatment of BC patients has been investigated in several small and non-randomized trials. It has been shown that response rates in patients treated with CPT derivatives in combination with various chemotherapeutic agents range from 14 to 64% [39]. This may reflect the wide heterogeneity of BC, as reflected in the high degree of diversity between and within tumors, as well as the high degree of diversity among cancer-bearing individuals. Decreased levels of TOP1 protein in BC cells have been associated with decreased sensitivity to CPT [40]; this finding is in line with data reported for colorectal cancer [41]. However, we previously demonstrated a direct correlation between TOP1 activity and the cellular drug response in various subpopulations of colon cancer cells that did not vary significantly with regard to TOP1 expression [26]. This observation implies that measurement of parameters other than TOP1 protein abundance or gene amplification across heterogeneous subpopulations of tumors may allow for prediction of the response to CPT.

BC cell lines are currently considered as valuable and informative models for generating functional criteria that can explain the drug sensitivity. Such criteria may be used for patient stratification and allow for more appropriate use of CPT in clinical trials [42–44]. However, no comprehensive examination has yet been performed to compare multiple parameters (including TOP1 activity and response to CPT) across cell lines corresponding to specific BC subtypes.

The aim of the current study was to characterize a set of BC cell lines (Luminal, HER2, and TNBC) according to basic functional parameters critical to the accumulation of CPT-induced TOP1cc and downstream effects. We measured seven genetic or metabolic characteristics of TOP1 that were considered to be most significant and informative for analysis of the functioning of the enzyme, including: (i) *TOP1* amplification status; (ii) TOP1 protein expression; (iii) TOP1 cleavage-ligation activity; (iv) susceptibility of TOP1 to CPT; (v) activity of tyrosyl-DNA phosphodiesterase 1 (TDP1); (vi) cellular response to CPT (cellular viability); (vii) cellular doubling time (growth rate).

## Methods

### Cell culture

A collection of human breast carcinoma cell lines (*n* = 24) was obtained from the American Tissue Culture Collection (ATCC) (Manassas, VA). Cells were cultured according to ATCC’s guidelines. SUM149 was purchased from Asterand Inc. (Detroit, MI, USA). Among the BC cell lines investigated, one (MCF10A) represented a normal-like mammary epithelial cell line [45]. Four were from patients who had amplified human epidermal growth factor receptor 2 (HER2+), representing the HER2-positive subtype (HCC1419, HCC1954, HCC202, and SK-BR-3). The two cell lines, HCC202 and HCC1954, are characterized by the lack of hormone receptor expression, including both ER and PgR. HCC1419 and SK-BR-3 express ER or ER and PgR, respectively (see Additional file 1: Table S1 for the details). They are both stratified as HER2 amplified Luminal B subtype cell lines. Five were from tumors without HER2 amplification, representing the Luminal BC subtype (BT474, HCC1428, MCF7, T47D, and ZR-75-1). Fourteen (BT549, CAL51, HCC38, HCC70, HCC1143, HCC1937, HCC1599, MDA-157, MDA-231, MDA-453, MDA-436, MDA-468, SUM149, and SUM1315) cell lines were from patients diagnosed with TNBC. The cells were grown until 70% confluency before collection. Additional file 1: Table S1 contains information for all cell lines propagated including receptor, known mutation status, ki67 status (reflects proliferation rate), and *TOP1* gene copy number [46]. Cellular growth rate was determined by measuring the average time required for a cell population to double in the log or exponential phase (i.e., during linear growth) [47]. All experiments were performed in duplicates.

### Quantification of TOP1 level by WB

Cells were harvested and counted with a Bürker-Türk chamber (Sigma-Aldrich, Denmark A/S). Aliquots of cellular pellets were stored at − 80 °C. Cellular pellets were lysed in NuPAGE LDS loading buffer and NuPAGE sample-reducing agent, followed by incubation at 70 °C for 10 min. Then 50 μg/lane (10^4^ cells) of protein extract was loaded onto NuPAGE Novex 4–12% Bis-Tris gels. Protein separation was performed in NuPAGE MOPS buffer containing NuPAGE antioxidant, according to the manufacturer’s instructions. WB was performed as described previously [48]. Resolved proteins were blotted onto Hybon-C nitrocellulose membranes (Amersham Biosciences, USA) and reacted with an hTOP1-specific rabbit antibody (1:2000; Epitomics, USA), followed by detection of immune complexes with a horseradish peroxidase-labeled polymer (1:200) (Envision+ detection kit; DAKO; Denmark). Blots were developed using the Amersham EC plus Western Blotting Detection Kit (GE Health/ Amersham Bioscience, VWR, Denmark), according to the manufacturer’s instructions. The blots were developed with mouse monoclonal anti-actin antibodies (1:2000; Santa Cruz, USA) as loading control. The intensities of TOP1 bands were normalized to the corresponding intensities of actin using PDQuest software (BioRad, USA) (see Additional file 1: Table S2). The quantification of TOP1 level by WB was performed based at both biological (× 2 times) and technical (× 2 times) replicates.

### IHC analysis

IHC analysis was performed as previously described [49]. Five-μm sections were cut from formalin-fixed paraffin-embedded (FFPE) cell blocks prepared as described previously [50] and mounted on Super Frost Plus slides (Menzel-Gläser, Braunschweig, Germany), then baked at 60 °C for 60 min, deparaffinized, and rehydrated through graded alcohol rinses. Heat-induced antigen retrieval was performed by immersing the slides in Tris/EDTA pH 9.0 buffer (10 mM Tris, 1 mM EDTA) and heating them in a 750-W microwave oven for 10 min. Slides were then cooled at room temperature for 20 min and rinsed in tap water. Non-specific staining of slides was blocked (10% fetal calf serum in PBS buffer) for 15 min, and endogenous peroxidase activity was quenched with 0.3% H_2_O_2_ in methanol for 30 min. Antigen was detected with antibodies raised against synthetic peptide corresponding to the N-terminus residues of human TOP1 (1:100, Epitomics, USA) and then by species-matched secondary antibody conjugated to a horseradish-peroxidase polymer (Envision+; DAKO, Glostrup, Denmark). We also used ki67 monoclonal mouse antibodies (MIB1 clone, DAKO, Glostrup, Denmark) at 1:200 to evaluate the rate of proliferation. Finally, color development was evaluated with 3, 3′- diaminobenzidine (Pierce, IL, USA) as chromogen to detect bound antibody complex. Slides were counterstained with hematoxylin. Dilution, incubation, and development times were standardized for accurate comparison of expression levels in all cases. Normal rabbit sera was used instead of primary antibody as a negative control.

### Reagents and enzymes

PrestoBlue reagent and Phi29 DNA polymerase were purchased from ThermoFisher Scientific. Streptavidin and all chemicals were purchased from Sigma-Aldrich, Denmark A/S.

### Oligonucleotide substrates, primers, and probes

Oligonucleotides for construction of the S (hTopI) substrate, TDP1 substrate, and amino primer (p) were obtained from DNA Technology A/S, Aarhus, Denmark and synthesized using the 394 DNA synthesizer from Applied Biosystems. Oligonucleotide sequences were as follows:

- S (TopI): 5’AGAAAAATTTTTAAAAAAACTGTGAAGATCGCTTATTTTTTTAAAAATTTTTCTAAGTCTTTTAGATC-CCTCAATGCACATGTTTGGCTCC-GATCTAAAAGACTT3’
- RCA primer: 5′ Am-CCAACCAACCAACCAAATAAGCGATCTTCACAGT3’
- TDP1 sensor: 6FAM-AAA GCA GGC TTC AAC GCA ACT GTG AAG ATC GCT TGG GTG CGT TGA AGC CTG CTT T-BHQ1, where 6FAM was attached to the DNA through a phosphothioate and to 3′BHQ1 through a phosphodiester linkage.

### Preparation of nuclear extract

Cells were harvested at the exponential phase of growth by brief treatment with 0.05% trypsin solution (Sigma-Aldrich, Denmark A/S) and counted with a Bürker-Türk chamber (Sigma-Aldrich, Denmark A/S). Cells were then centrifuged (300×*g*), and cell pellets were frozen at − 80 °C. To prepare nuclear extracts (used in all REEAD experiments), cells were lysed in buffer B (0.1% NP-40, 10 mM Tris, pH 7.9, 10 mM MgCl_2_, 15 mM NaCl, 1 mM DTT) and 0.1 mM phenylmethylsulfonyl fluoride (PMSF) on ice for 10 min. Nuclei were then pelleted by centrifugation at 400×*g* for 10 min. Pelleted nuclei were extracted by addition of 100 μl nuclear extraction buffer (0.5 M NaCl, 20 mM HEPES, pH 7.9, 20% glycerol, 1 mM DTT, and 0.1 mM PMSF), followed by rotation for 1 h at 4 °C [51]; fresh PMSF was added every 15 min. Cell debris was removed by centrifugation at 9000×*g* for 10 min at 4 °C.

### Detection of TOP1 activity by REEAD assay

The REEAD assay was performed essentially as previously described [26, 52] TOP1 reactions were carried out in a 10-μL reaction volume containing divalent cation depletion buffer (10 mM Tris-HCl, pH 7.5, 5 mM EDTA, and 50 mM NaCl). Reaction mixtures were supplemented with 500 nM S (TopI) DNA substrate. Reactions were initiated by the addition of 1 μL of nuclear extract (obtained from the same cell samples used for quantification of TOP1 protein level by WB) or purified TOP1 (previously prepared and used for all experiments as positive control). Incubation was continued for 30 min at 37 °C before heat inactivation of enzymes present in the reaction mixture for 5 min at 95 °C. Circularized closed circles were then incubated with 5 μM of primer and 5 units of Phi29 polymerase in Phi29 buffer, in the presence of 0.1 mM dNTP with 10% biotinylated dCTP and alpha ^P^32 dATP. After 2 h at 37 °C, 7.5 μg of streptavidin was added, and reactions were blotted on a nitrocellulose membrane already soaked in blotting buffer (25 mM Tris-HCl, 192 mM glycine, 0.1% SDS, pH approx. 8.6, 10% EtOH buffer). Membranes were washed 3 × 10 minutes with wash buffer 1 (20 mM Tris-HCl pH 7.5, 50 mM NaCl, 0.2% Tween20) and 3 × 10 minutes with wash buffer 2 (0.30 M NaCl, 0.030 M trisodium citrate). After 5′ minutes of air-dry, the membrane was exposed in a Phosphorimager cassette overnight. Intensity of the radioactive dots was quantified using QuantityOne software. For all cell lines, TOP1 activity was normalized to activity of purified TOP1 circles and protein concentration in nuclear extracts as determined with Bradford’s protocol Results were plotted as average of three independent experiments using GraphPad Prism Software.

### Cell viability assay

Cell viability after CPT treatment was determined with PrestoBlue cell viability reagent (Invitrogen), according to the manufacturer’s instructions. A 20 mM stock of CPT was prepared in DMSO; all working dilutions were prepared in cell media. Cells (amount determined by growth rate) were seeded with 100 μl growth medium into 96-well flat-bottom plates (Corning, Inc., Corning, NY) and incubated for 24 h. After 24 h, cell media were replaced with medium alone, medium with DMSO, or medium with CPT (concentration ranging from 0.1 μM to 5 μM). Untreated cells, cells incubated with DMSO, and cells treated with CPT (12 wells per treatment) were incubated for 72 h. At the end of treatment, 10 μl of PrestoBlue cell viability reagent was added. Samples were then incubated for 4 h in the dark at 37 °C. Fluorescence emission was measured at 540-nm excitation/590-nm emission using FLUOstar OPTIMA microplate reader (BMG Labtech, Ortenberg, Germany). DMSO concentration was corrected to 0.5% in all wells. After background subtraction, fluorescence values were normalized to the highest fluorescence measured in any well, which corresponded to the value for the cell well without treatment. Data were plotted as mean (12 wells) ± SD values using GraphPad Prism Software.

### CPT susceptibility of TOP1 in nuclear cell extract

Sensitivity of TOP1 to CPT in nuclear cell extracts was measured by REAAD and analyzed as a radiolabeled-dot-blot readout, as described for the measurement of TOP1 activity. Nuclear cell extract was prepared as mentioned above and incubated in cation-depletion buffer without S (TopI) (in DMSO as a positive control) or with CPT (15 μM, 30 μM, or 60 μM), as stated in Additional file 1: Figure S8. Samples that did not contain nuclear extract were used as negative control. The reactions were carried out for 10 min at room temperature, then stopped at 95 °C for 5 min. Circularized closed circles were then incubated with primer, amplified by rolling circle amplification, and blotted on a nitrocellulose membrane, as stated above. Intensity of the radiolabeled dots was normalized to the intensity of DMSO dots for each cell line. Plotted results represent mean ± SD. TOP1 susceptibility to CPT was investigated in selected cell lines at the highest concentration of inhibitor (− 60 μM), as depicted in Fig. 4a.

### TDP1 activity

TDP1 activity measurements were carried out using a final concentration of 0.5 μM TDP1-sensor in a volume of 25 μL containing 1xTDP1 buffer (20 mM Tris– HCl pH 8, 100 mM KCl, 10 mM EDTA, 10 mM DTT, and 0.05% Triton X-100) supplemented with a dilution series of cell extract. Reactions were carried out at 37 °C in a real-time PCR machine; fluorescence intensity was measured every 30 s. Data were imported into Excel, and slope during the linear phase (here 15–30 min) was calculated for each dilution of cell extract. Purified TDP1 [53] was used as positive control.

### Statistical analysis

Data were analyzed using GraphPad Prism software and expressed as mean ± SD. Linear regression was performed, and correlation analysis was conducted with Spearman’s correlation test.

## Results

### TOP1 protein expression and gene amplification

**Fig. 1:**
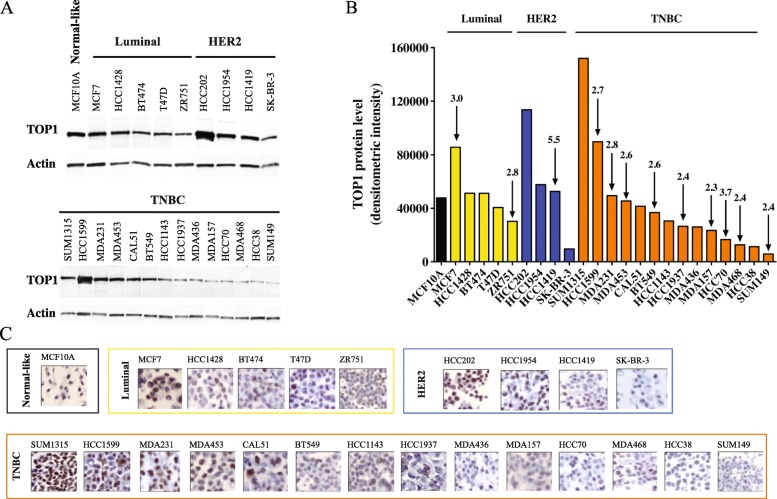
Quantitative analysis of TOP1 protein expression and TOP1 gene amplification across BC cell lines. **a** Western blot images. Blots were developed first with antibodies against TOP1 and then with anti-actin antibodies. BC cell line subtypes are indicated at the tops of the images. **b** The graph shows normalized data from (**a**). TOP1 protein levels in various BC cell lines were normalized to actin levels (see Methods). The arrows indicate *TOP1* copy number in each cell line with amplification [46]. BC cell line subtype-based classification is indicated by bars and by colors: *black* – normal-like MCF10A cell line; *yellow* - Luminal subtype; *blue* - HER2 amplified, and *orange* - TNBC subtype. **c** Representative IHC images show TOP1 expression across BC cell lines. IHC sections (× 20 magnification) are placed in the same order as in **a** and **b**. For details, see magnified images (× 40) of nuclear TOP1 IHC staining of all BC cell lines analyzed in Additional file 1: Figure S1

Quantitative western blot (WB) was performed to measure relative protein expression of TOP1 across the panel of cell lines representing the major breast tumor subtypes (Fig. 1a-b, Additional file 1: Table S2). We sought to determine whether TOP1 protein expression correlated with *TOP1* amplification, as previously observed [46]. The cell panel used in our study consists of a MCF10A cell line representing non-tumorigenic breast epithelial cells and cell lines that originated in patients bearing Luminal (5 cell lines), HER2 (4 cell lines), or TNBC (14 cell lines) tumors. As seen from the data shown in Fig. 1a-b, relative protein expression of TOP1 varied strongly across cell lines, even lines representing the same BC subtype. The results of immunohistochemistry (IHC) staining showed near-exclusive intracellular localization of TOP1 within nuclei (Fig. 1c and Additional file 1: Figure S1).

*TOP1* was amplified in two of five Luminal cell lines tested, in one of four HER2 cell lines, and in nine of 14 TNBC cell lines (Fig. 1b; cell lines with gene amplification are indicated with arrows). The Luminal MCF7 cell line, in which *TOP1* is amplified threefold, is characterized by an increase in TOP1 protein expression of approximately twofold, compared with levels observed in the HCC1428, BT474, and T47D cell lines (Fig. 1b). However, the association observed for these three cell lines of Luminal subtype cannot be considered as strong evidence that elevated TOP1 expression is caused solely by gene amplification. Indeed, among cell lines of the Luminal subgroup, the ZR751 cell line exhibited the lowest level of TOP1 protein expression, with *TOP1* copy number similar to that observed in MCF7. Spearman correlation analysis of a linear regression performed for all Luminal cell lines did not reveal any correlation between TOP1 protein expression and *TOP1* copy number (*R*^2^ = 0.1169, p (one tailed) = 0.2535, Additional file 1: Figure S2).

The two other groups of cell lines, representing subtypes HER2 and TNBC, respectively, also failed to reveal a significant correlation between *TOP1* gene status and protein expression of TOP1 (*R*^2^ = 0.007914, p (one tailed) = 0.4555, Additional file 1: Figure S3; *R*^2^ = 0.04378, p (one tailed) = 0.2364, Additional file 1: Figure S4, respectively). Among the HER2 cell lines, HCC202 showed the highest level of TOP1 protein expression, without any *TOP1* amplification. HCC1419, which had the highest *TOP1* copy number (5.5), exhibited approximately the same level of TOP1 protein expression as HCC1954, without amplification of the *TOP1* gene. The lack of correlation between *TOP1* copy number and amount of TOP1 protein expressed also applied to the TNBC subtype. Overall, our data clearly demonstrated high variability in TOP1 expression levels across BC cell lines and the absence of a significant correlation between *TOP1* copy number and protein expression. Interestingly, some of the cancer cell lines appeared to be characterized by a lower TOP1 protein amount than the normal like MCF10A cell line. This could rise questions to the rationale of using TOP1 as a target in cancer treatment. However, here it is important to remember, 1) it is the TOP1 activity and CPT susceptibility that the determine the number of TOP1cc generated, 2) it is the conversion of TOP1cc to lethal DNA damage that determine the cytotoxicity of CPT. These factors may not necessarily depend on TOP1 protein amount per se.

### TOP1 enzymatic activity

To identify a presumptive association between TOP1 protein expression and intracellular enzymatic activity among cell lines representing three BC subtypes, the latter was measured in nuclear extracts with REEAD [52]. This technique provides a highly sensitive and quantitative measurement of enzymatic activity in whole-cell or nuclear extracts by specifically monitoring the cleavage-ligation step that is the critical target for CPT interference.

**Fig. 2:**
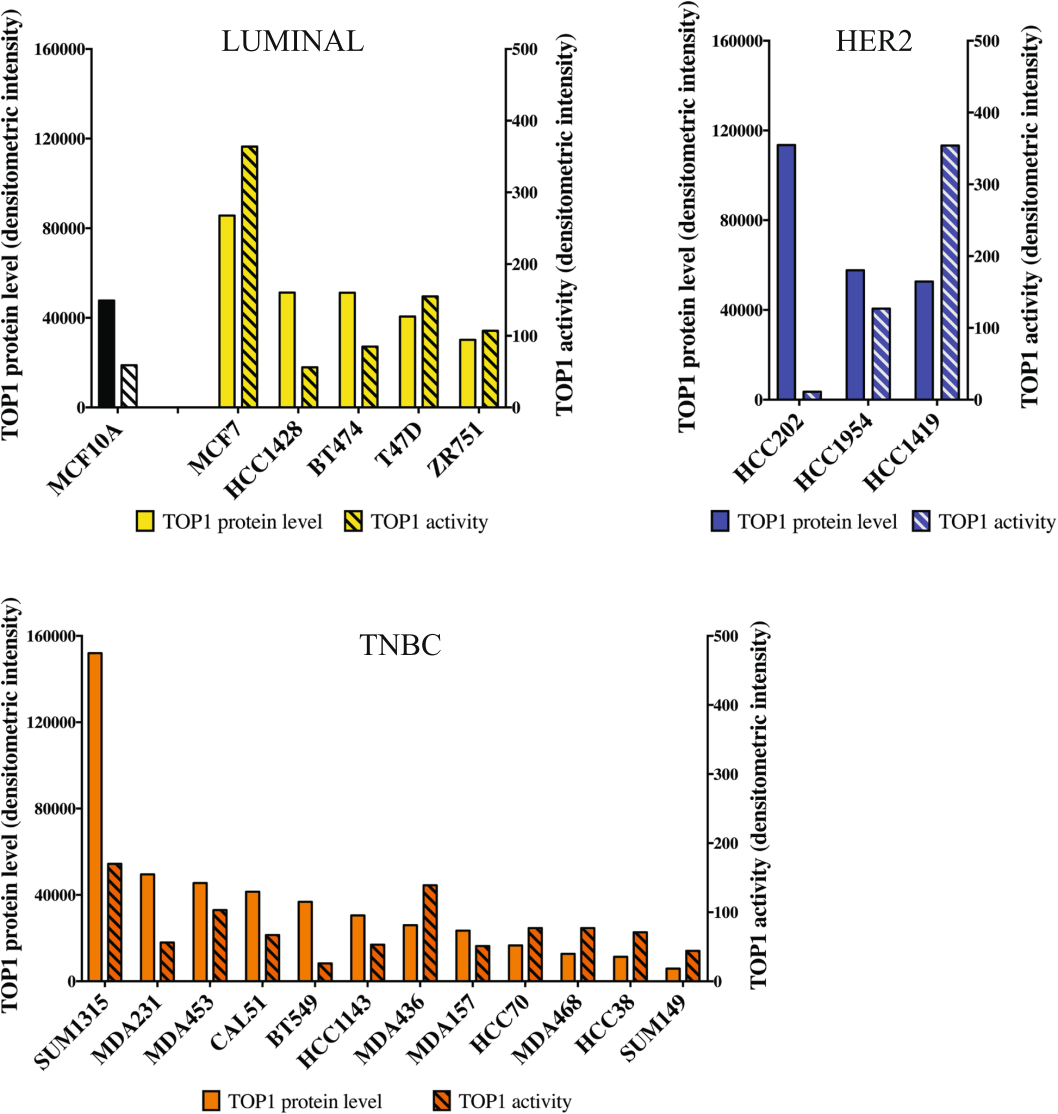
Comparative analysis of cleavage-ligation TOP1 activity and corresponding protein level. Bar-charts show TOP1 activity (lined bars) in nuclear extracts obtained from each cell line as measured by REEAD and TOP1 protein level (full bars) as determined by western blot (see Fig. 1b). TOP1 activity is shown as an average of three independent experiments. The order of cell line distribution across the “X” axis is the same as in Fig. 1. Yellow - Luminal; blue - HER2; orange - TNBC

Figure 2 shows the levels of TOP1 enzymatic activity, in relation to corresponding TOP1 protein levels (see Fig. 1a-b as reference), across 21 cell lines. Spearman correlation analysis of a linear regression of the data presented in Fig. 2 revealed a significant correlation between enzymatic activity and protein TOP1 expression in Luminal-type (*R*^2^ = 0.6093, *p* = 0.0335; Additional file 1: Figure S5) as well as TNBC-like cell lines (*R*^2^ = 0.4392, *p* = 0.0094; Additional file 1: Figure S6). No correlation between TOP1 protein expression and activity levels was observed in HER2 cell lines (*R*^2^ = 0.6488, *p* = 0.2019; Additional file 1: Figure S7). However, this could be due to the limited number of HER2 cell lines available for analysis.

### Measurement of the cellular response to CPT

**Fig. 3:**
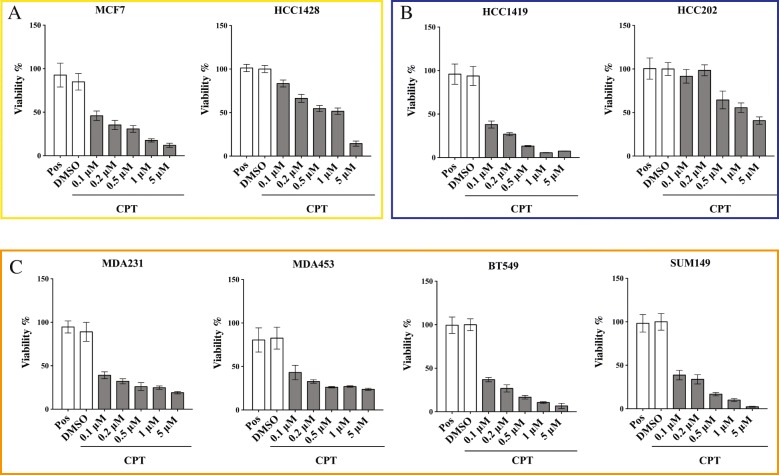
The response to CPT among eight selected cell lines with various levels of TOP1 enzyme activity. The cell lines indicated were treated with DMSO (as baseline control) or increasing concentrations of CPT (0.1 to 5 μM). Cellular viability was then measured using PrestoBlue reagent. The viability of the cells in absence of any treatment was included as positive control (Pos). Fluorescence signals were normalized to the positive control in each cell line, as described in Materials and Methods. Data plotted are mean (*N* = 12) ± SD. **a** Luminal subtype cell lines, **b** HER2 subtype cell lines, **c** TNBC subtype cell lines

To address whether intracellular TOP1 activity correlates with the cellular response to CPT, we selected a subset of cell lines representing each BC subtype for viability studies. When choosing cell lines, we were guided by the criterion of diversity. Hence, cell lines with high or low TOP1 activity level, as determined in Fig. 2 (“high” = densitometric intensity > 350, “low” = densitometric intensity < 100 units) were selected. The cell lines selected were as follows: Luminal subtype MCF7 (high TOP1 activity), HCC1428 (low TOP1 activity), HER2 subtype HCC1419 (high TOP1 activity), and HCC202 (low TOP1 activity). Since none of the TNBC cell lines analyzed in this study showed high TOP1 enzymatic activity (see Fig. 2), we selected four TNBC cell lines with diverse levels of TOP1 activity, ranging from 110 to 20 densitometric intensity units (MDA453, MDA231, Sum149, and BT549). CPT sensitivity was measured using a standard survival assay based on the treatment of cells with concentrations of CPT ranging from 0.1 μM to 5 μM for 72 h, followed by staining with PrestoBlue (Thermofisher Scientific) [26, 54]. Untreated cells as well as cells treated with DMSO (solvent used for CPT) were included in the experiment as positive controls (see Methods). The results are presented in Fig. 3. Half-maximal inhibitory concentrations (IC_50_) were estimated using GraphPad Prism software (Table 1).

**Table 1:** Gene copy number, cellular viability and TOP1 enzymatic activity in selected cell lines

Cell line	BC subtype	TOP1 Gene copy number	Viability IC50 (μM)	TOP1 Enzymatic activity
MCF7	Luminal	3	0.089 ± 0.017	High
HCC1428	Luminal	No amplification	0.448 ± 0.054	Low
HCC202	HER 2	No amplification	0.481 ± 0.060	Low
HCC1419	HER 2	5.5	0.067 ± 0.010	High
MDAB231	TNBC	2.8	0.040 ± 0.011	Low
MDAMB453	TNBC	2.6	0.058 ± 0.019	Low
BT549	TNBC	2.6	0.056 ± 0.007	Low
SUM149	TNBC	2.4	0.065 ± 0.010	Low

The two cell lines with high TOP1 enzymatic activity [MCF7 (Luminal subtype) and HCC1419 (HER2 subtype)] showed high sensitivity toward CPT treatment, exhibiting an IC_50_ of 0.089 ± 0.017 μM and 0.067 ± 0.010 μM, respectively. Cell lines with low TOP1 activity [HCC1428 (Luminal subtype) and HCC202 (HER2 subtype)] exhibited greater resistance to the drug, with IC_50_ of 0.448 ± 0.054 μM and 0.481 ± 0.060 μM, respectively (Table 1). These results show a direct correlation between TOP1 activity and cellular response to CPT in cell lines from the Luminal and HER2 subtypes. However, the significance of the correlation could not be confirmed due to the limited number of samples available for Spearman analysis. The observed correlation is in agreement with the CPT’s mechanism of action [17, 55]. Cell lines derived from the TNBC subtype, however, did not appear to follow this pattern. Cell lines derived from the TNBC subtype had low TOP1 enzymatic activity and showed high sensitivity toward CPT, with IC_50_ < 0.07 μM (Table 1). Spearman correlation applied to the entire group of selected cell lines without regard to their subtype did not show a significant correlation between cellular response to CPT and TOP1 enzymatic activity level (data not shown). It is worthwhile to note, however, that all TNBC cell lines were characterized by high CPT sensitivity, regardless of the mechanism behind this sensitivity.

### TOP1 susceptibility toward CPT and TDP1 activity in BC cell lines

**Fig. 4:**
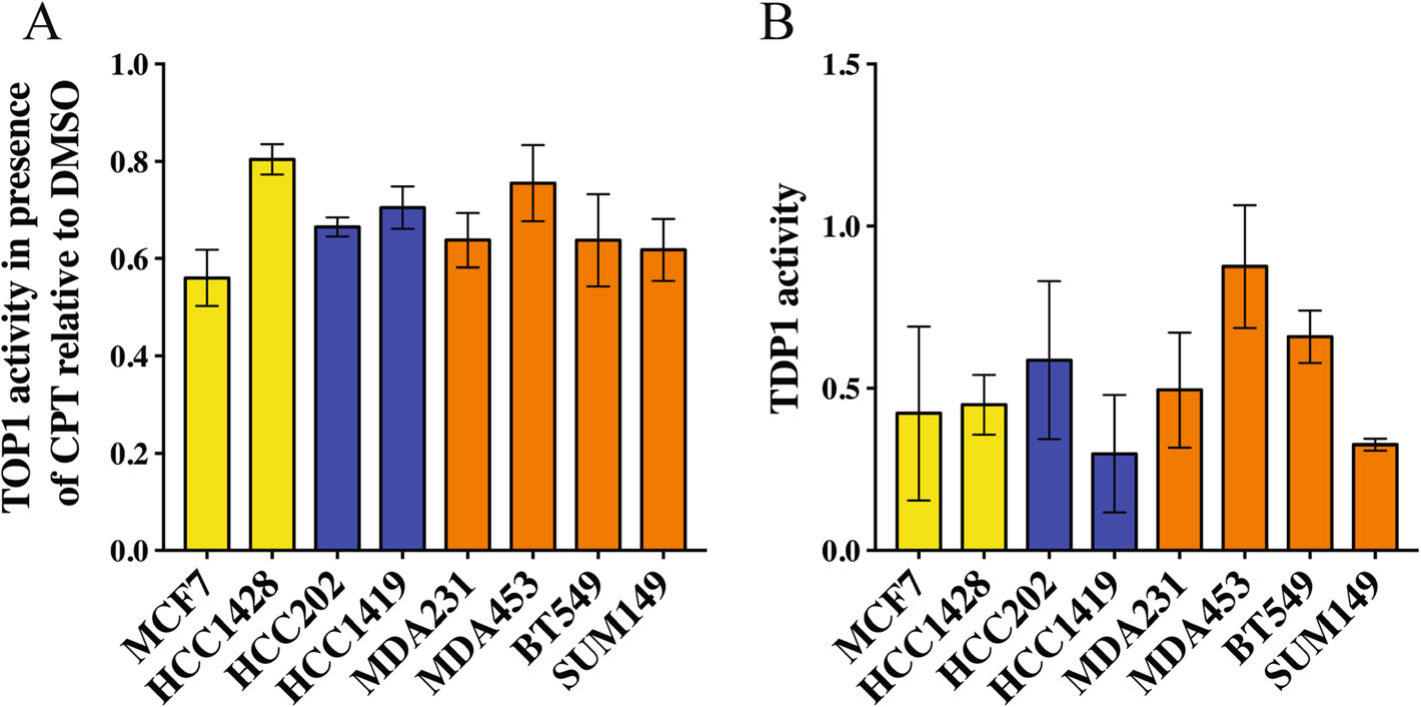
Susceptibility of TOP1 cleavage-ligation to CPT and TDP1 in nuclear extracts for eight selected BC cell lines. **a** Efficiency of the TOP1 cleavage-ligation step was measured in nuclear extracts isolated from selected cultured cells after incubation with DMSO (control) or 60 μM CPT using the REEAD assay. Intensity of the REEAD signal obtained when measuring TOP1 activity in the presence of CPT was normalized to the signal obtained in DMSO for each cell line (REEAD signal in CPT/REEAD signal in DMSO). Results were plotted as mean ± SD (four independent experiments). Yellow bars - Luminal cell lines; blue bars - HER2 cell lines; orange bars - TNBC cell lines. **b** Bar charts of TDP1 activity measured in cell extracts from selected cell lines. TDP1 activity measured in terms of fluorescence signals was plotted as average ± SD (three independent experiments). Yellow bars - Luminal cell lines; blue bars - HER2 cell lines; orange bars - TNBC cell lines

In addition to TOP1 activity, the drug susceptibility of the enzyme itself can influence cellular sensitivity to CPT. The susceptibility of TOP1 toward CPT was estimated by measuring TOP1 activity in nuclear extracts prepared from the eight cell lines selected in the presence of 60 μM CPT, using the REEAD assay. Note that CPT susceptibility as measured in terms of inhibition of the (nuclear-extracted) TOP1 re-ligation activity is not dependent on TOP1 activity per se (previously measured, see Fig. 2). The results are graphically depicted in bar-charts in Fig. 4a as the ratio of REEAD signal obtained in the presence of 60 μM CPT relative to the REEAD signal obtained in the presence of DMSO. The results of similar analyses performed with titration of CPT are shown in Additional file 1: Figure S8).

As seen from the bar-charts (Fig. 4a and Additional file 1: Figure S8), analysis of nuclear extracts revealed no significant difference among cell lines in terms of TOP1 susceptibility toward CPT. The level of TOP1 activity observed at even the highest concentration used (60 μM) of CPT varied from 60 to 80% relative to the DMSO control. These results demonstrate that all cell lines analyzed in this experiment are characterized by approximately the same degree of enzyme susceptibility to the drug. We found no significant correlation between TOP1 susceptibility toward CPT and the cellular response to CPT (cellular viability, data not shown).

Another potential molecular determinant of cellular response to CPT is the ability of the cellular machinery to repair DNA damage induced by CPT. TDP1 is a key enzyme that participates in the removal and repair of TOP1cc in human cells [30]. To assess a possible association between the cellular response to CPT and TDP1 activity, the latter was measured in nuclear extracts of selected cell lines using a real-time DNA sensor assay previously developed by our group ([53], Fig. 4b). Since TDP1 repairs CPT-induced DNA damage, a reverse correlation between cellular response to CPT and TDP1 activity was expected. However, we did not observe such a correlation. In fact, TDP1 activity varied strongly among cell lines. Hence, TDP1 activity alone cannot explain the differential CPT sensitivity of the BC cell lines investigated.

### Correlation between cellular response to CPT and cellular growth rate

**Fig. 5:**
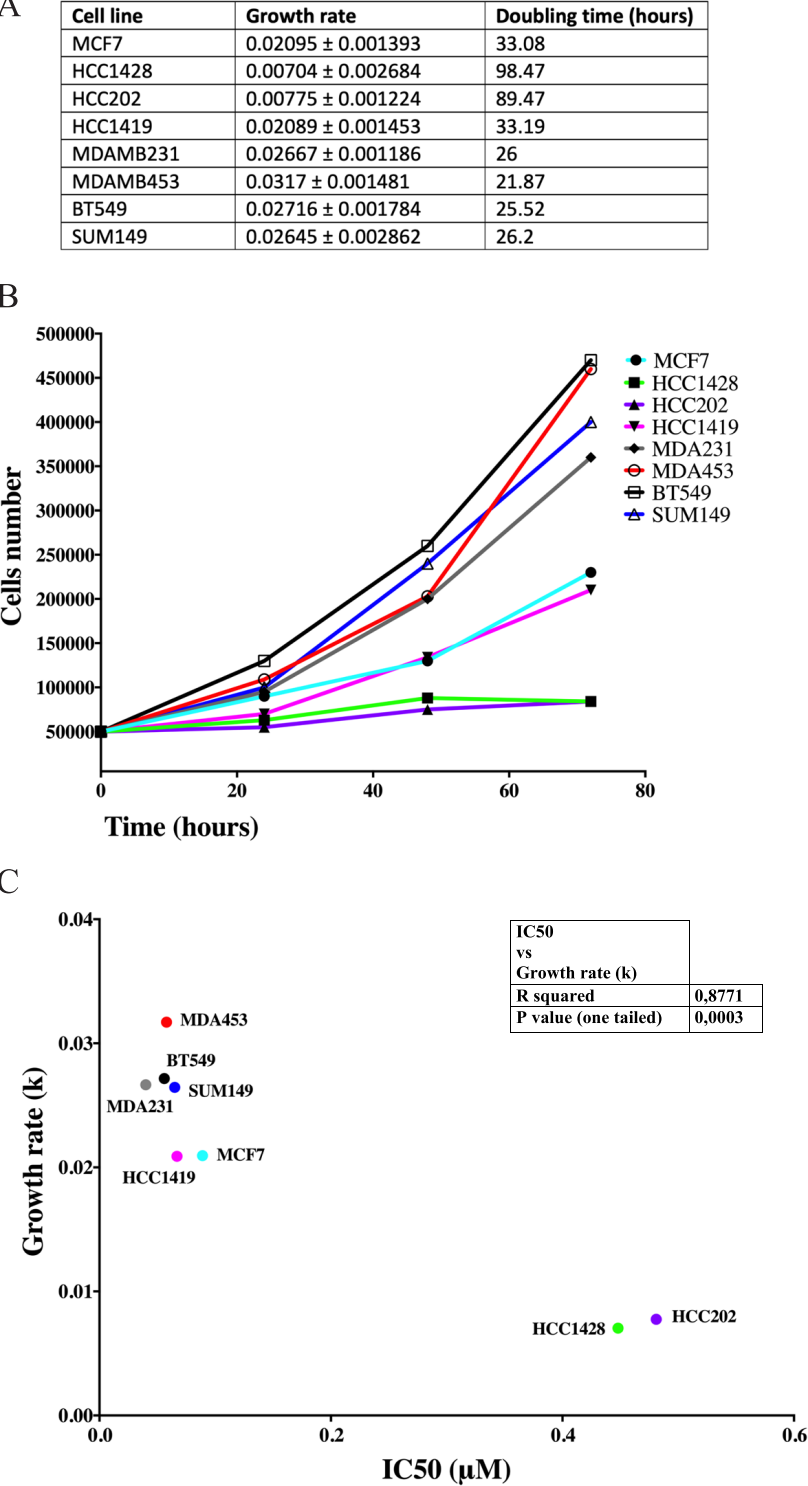
Growth rate for eight cell lines and correlation between growth rate and IC_50_. **a** Table shows the estimated growth rate and doubling time for Luminal cells (MCF7 and HCC1428), HER2 subtype (HCC1419 and HCC202), and TNBC subtype (MDA453, MDA231, SUM149, and BT549). Cellular doubling time was determined by evaluation of the time that it takes for a cell population to double during the log-phase phase (i.e., during linear growth). **b** Graphical depiction of cellular proliferation over time. The number of cells measured at each time point (x-axis) is specified at the y -axis. **c** Graphical depiction of the correlation between IC_50_ and growth rate (k). Yellow dots - Luminal cell lines; blue dots - HER2 cell lines; orange dots - TNBC cell liness

As mentioned above, TOP1cc stabilized by CPT are converted to permanent double-strand DNA breaks by collision with replication forks [18]. Consequently, it can be assumed that fast-dividing cells may be more drug sensitive than slowly dividing cells. We therefore investigated whether the differential drug responses observed in BC cell lines correlated with cellular growth rate. The latter parameter was evaluated by measuring doubling time, i.e., the average time it takes a cell population to double during the log-phase, as described elsewhere [47]. The results are shown in the table in Fig. 5a and plotted in Fig. 5b. Using the IC_50_ parameter, we found a significant direct correlation between growth rate and cellular response to CPT in all cell lines investigated (Fig. 5c: *R*^2^ = 0.8771, p (one tailed) = 0.0003). Hence, all four TNBC-like cell lines (MDA453, MDA231, BT549, SUM149) had a high growth rate (Fig. 5) and were very sensitive to CPT (low IC_50_, Figs. 3c and 5c). The same trend was also observed in Luminal and HER2 cell lines (Fig. 5c). The MCF7 (Luminal) and HCC1419 (HER2) cell lines that exhibited a strong response to CPT (low IC_50_, Figs. 3a, b and 5c) divided much faster than counterparts with low sensitivity to CPT (high IC_50_; HCC1428 and HCC202) (Figs. 3a, b and 5c).

## Discussion

The issues that spurred our study included the limited number of drugs currently available in clinical practice for effective and personalized therapy of multiple BC subtypes and development of acquired resistance to treatment. The aim was to provide detailed insight into CPT drug susceptibility across cell lines representing major types of BC. CPT associated derivatives that target TOP1 are currently applied as second or third lines of treatment for endocrine resistant BC patients. As mentioned, CPT acts by leading to the accumulation of TOP1cc. Such damage is lethal when encountered by a replication fork [18]. The efficiency and cytotoxic effect of the CPT may be regulated by other means than TOP1 expression and activity. Hence, the large variation in CPT response rates observed in small and non-randomized trials for BC may be due, at least in part, to a lack of reliable selective parameters for patient stratification [56].

BC cell lines provide unlimited homogeneous material for modelling studies designed to investigate disease pathobiology as well as to identify novel targets for therapeutic intervention. In the present study, we evaluated TOP1 status in a representative panel of cell lines derived from three main BC subtypes (Luminal, HER2, and TNBC). The aim was to investigate possible associations of cellular response to CPT with various cellular and molecular factors, including *TOP1* copy number, TOP1 protein expression, enzyme activity, TOP1 susceptibility to CPT, TDP1 activity, and cellular growth rate. The results are summarized in Additional file 1: Table S3.

It has been shown that a gain of the *TOP1* gene is common in patients with BC and is often considered as a potential biomarker of response to treatment with TOP1 inhibitors [39, 57, 58]. In our study, *TOP1* was found to be amplified in two of five Luminal cell lines, one of four HER2 cell lines, and nine of 14 TNBC-like cell lines ([46] Fig. 1). Our data showed high variability in levels of TOP1 protein across all analyzed BC cell lines. However, the results obtained did not indicate a significant correlation between protein level and *TOP1* copy number, as was estimated by Spearman analysis for the three individual groups of BC cell lines (Fig. 1, Additional file 1: Figures S2-S4). The lack of a significant association between gene copy number and protein level observed in the present study contrasts with results reported previously by McLeod and Keith, who analyzed four BC cell lines (MCF7, ZR751, MDA231, and MDA436). The authors reported a significant relationship between TOP1 copy number and protein level [35]. This inconsistency may reflect the small number of cell lines analyzed by McLeod and Keith (three cell lines with amplification of the *TOP1* locus and a single cell line without *TOP1* amplification). Jandu and co-authors reported a putative association between TOP1 protein level and gene copy number in nine BC cell lines, but it was not possible to determine the significance of this presumptive correlation, because the level of TOP1 protein expression was evaluated by eye rather than by quantitative densitometry [46]. In general, variations in protein expression of TOP1 in BC cell lines may be caused by amplification of the *TOP1* locus or by other aspects of cell metabolism. Current data regarding *TOP1* gene status in breast tumor tissue are rather controversial. An investigation of *TOP1* gene copy number (including 1033 cases) based on use of the The Cancer Genome Atlas (TCGA) portal revealed that only 2% of breast tumors exhibited increased *TOP1* copy number [57]. However, FISH analysis of cancer cells showed a much higher proportion of *TOP1* amplification (> 30% of BC patients had gene copy numbers > 4). This discrepancy in the overall analysis of gene copy number may be explained by a dilution effect caused by the presence of normal stromal cells [57]. The question of the extent to which the gene status of BC cell lines can simulate the situation in tumor tissue remains problematic and requires further investigation. In addition, it should be mentioned that any cellular model system that is used for the search of parameters that reflect intra-cellular drug responsiveness is unable to reproduce the whole complexity of a human tumor and to predict potential impact of intra-tumor heterogeneity that plays an essential role in tumor development and drug responsiveness. Those factors, i.e. the presence of multiple clones within a single tumor or multiple cell types presented within tumor microenvironment, would be especially essential in TNBC tumors that are often characterized by high degree of complexity and clonal heterogeneity [12].

We observed a direct association between protein level and cleavage-ligation activity of TOP1 in Luminal and TNBC-like subtypes but not for the HER2 subtype. The lack of a significant correlation for HER2-like cells may be partially explained by the small number of cell lines of this subtype available for experimentation or, alternatively, by specific cellular metabolic features associated with HER2 amplification [59].

Given the labor-intensity of experiments involving the analysis of multiple parameters (cellular response to CPT, susceptibility of TOP1 to CPT, analysis of TDP1 activity, and cellular growth rate) across numerous cell lines, we selected eight cell lines that represented three main BC subtypes. A Spearman correlation test applied to all cell lines investigated showed that there was no correlation between *TOP1* gene copy number, protein expression, enzyme activity, and CPT-induced cytotoxicity. This is in contrast to results obtained from cell lines originating from tumors other than breast cancer, which demonstrated a direct correlation between *TOP1* gene copy number and IC_50_ [60–62]. However, data published by other groups on BC-derived cell lines are conflicting [35, 46]. For cell lines derived from Luminal and HER2 subtypes, we did see a correlation between TOP1 activity and CPT-induced cytotoxicity, but our data were insufficient for statistical analysis. Further studies on a broader panel of Luminal and, in particular, HER2 cell lines are required to confirm a correlation between the parameters measured. However, such a study could be hindered by the limited number of BC cell lines (especially HER2-subtype lines) that have been generated and properly characterized to date [44].

In addition to the TOP1 activity, drug susceptibility of the enzyme itself may influence cellular sensitivity to CPT [19, 55]. We previously demonstrated a direct correlation between CPT susceptibility to TOP1 and the cellular drug response in two subpopulations of colon cancer-derived Caco2 cells [26]. All eight BC cell lines analyzed in this study showed, however, similar TOP1 susceptibility toward the drug and we did not find a significant correlation between the CPT susceptibility of TOP1 and the cellular response to CPT (cellular viability).

TDP1 is an enzyme that catalyzes the repair of several types of DNA damage, including CPT-induced DNA damage [29, 63]. Consequently, a reverse correlation between cellular response to CPT and TDP1 activity can be expected. However, comparison of selected cell lines did not reveal a significant correlation between these parameters (data not shown). Therefore, TDP1 activity cannot explain the differential CPT sensitivity observed among BC cell lines. Most likely, the effect of TDP1 activity cannot be regarded as a single factor, and several repair pathways complement TDP1 activity [64]. Investigations of all possible repair pathways were not within the scope of the current study.

It is well established that when a replication fork is encountered by CPT-stabilized TOP1cc, DNA fragmentation and cell death follow. It can be assumed that rapidly dividing cells are more drug sensitive than slowly dividing cells. We therefore investigated whether the differential drug response observed among the cell lines analyzed correlated with growth rate. When dividing the cell lines into two broad categories slow growing (double time above 89 h) and fast growing (doubling time below 34 h) we identified a direct association between the rate of cell division and sensitivity to CPT. This finding is in agreement with previous studies reporting that the cell-killing mechanism of CPT is S-phase dependent [23, 65]. Our results are also consistent with a recently published study showing that BC cell lines with acquired or de novo resistance to the CPT derivative SN-38 had significantly (*p* < 0.05) lower growth rates, when compared to their parental and DMSO controls [46].

It is striking to note that four of the cell lines (MDA453, MDA231, BT549, SUM149) characterized by a high growth rate and high CPT sensitivity have low TOP1 activity. This may suggest off target effects of CPT, but is more likely explained by the fact that the cytotoxicity of CPT depend on collision of replication forks with CPT induced TOP1cc resulting in the generation of lethal DNA damage. It is possible that in very fast-growing cells even a low activity of TOP1 is sufficient to generate sufficient TOP1cc in the presence of CPT for efficient cell killing. Indeed, in cells with defect repair pathways, which is a key mark of cancer cells, even a single TOP1cc at the right place and time may kill the cells [66]. Hence, the observed results may very well be accommodated in the current model of replication depended cytotoxicity of CPT.

## Conclusions

Taken together, our results suggest that *TOP1* gene copy number is a poor indicator for CPT response in BC cell lines. More comprehensive studies are needed to confirm this hypothesis for each breast cancer subtype. Indeed, for cell lines derived from the HER2 and Luminal subtypes, our data point to a putative direct correlation between gene copy number, TOP1 activity, and cellular sensitivity to CPT, suggesting that TOP1 at either the genomic or enzymatic level may be a predictive marker for response to CPT in these BC subtypes. For all BC subtypes, there is a direct correlation between growth rate and cellular response to CPT. Collectively, our results suggest that more aggressive and quickly dividing malignant tumors may be targeted more effectively by CPT. The extremely powerful response of cell lines derived from TNBC tumors treated with CPT appears promising, suggesting that it may be worthwhile to investigate the possibility of treating patients with this subtype of BC with CPT derivatives.

## Supplementary information

**Additional file 1: Table S1.** description of the cell lines used in the study. **Table S2.** Shows the result of the quantifications of the TOP1 protein levels. **Figure S1.** Nuclear TOP1 IHC staining. **Figure S2.** Shows the correlation between TOP1 protein expression level and TOP1 gene copy number in cell lines of the luminal subtype. **Figure S3.** Shows the correlation between TOP1 protein expression level and TOP1 gene copy number in cell lines of the HER2 subtype. **Figure S4.** Shows the correlation between TOP1 protein expression level and TOP1 gene copy number in cell lines of the TNBC subtype. **Figure S5.** Shows the correlation between TOP1 activity and protein expression level in cell lines of the luminal subtype. **Figure S6.** Shows the correlation between TOP1 activity and protein expression level in cell lines of the TNBC subtype. **Figure S7.** Shows the correlation between TOP1 activity and protein expression level in cell lines of the HER2 subtype. **Figure S8.** Shows a graphically depiction of TOP1 susceptibility to CPT in nuclear extracts. **Table S3.** Summary of the results obtained for all investigated parameters.

## Data Availability

The data analyzed during the current study are available from the corresponding author on reasonable request.

## Abbreviations

BC: Breast cancer
ER: Estrogen receptor
FFPE: Formalin-fixed paraffin-embedded
IHC: Immunohistochemistry
PgR: Progesterone receptor
REEAD: Rolling circle enhanced enzyme activity detection
TNBC: Triple-negative breast cancer
