# Supplementary material for: Topoisomerase I activity and sensitivity to camptothecin in breast cancer-derived cells: a comparative study
Source: BMC Cancer. 2019 Nov 29;19:1158. doi: 10.1186/s12885-019-6371-0 (PMC6884793; doi:10.1186/s12885-019-6371-0)
Supplement: Supplementary file 1 — Additional file 1: Table S1. description of the cell lines used in the study. Table S2. Shows the result of the quantifications of the TOP1 protein levels. Figure S1. Nuclear TOP1 IHC staining. Figure S2. Shows the correlation between TOP1 protein expression level and TOP1 gene copy number in cell lines of the luminal subtype. Figure S3. Shows the correlation between TOP1 protein expression level and TOP1 gene copy number in cell lines of the HER2 subtype. Figure S4. Shows the correlation between TOP1 protein expression level and TOP1 gene copy number in cell lines of the TNBC subtype. Figure S5. Shows the correlation between TOP1 activity and protein expression level in cell lines of the luminal subtype. Figure S6. Shows the correlation between TOP1 activity and protein expression level in cell lines of the TNBC subtype. Figure S7. Shows the correlation between TOP1 activity and protein expression level in cell lines of the HER2 subtype. Figure S8. Shows a graphically depiction of TOP1 susceptibility to CPT in nuclear extracts. Table S3. Summary of the results obtained for all investigated parameters. [file 12885_2019_6371_MOESM1_ESM.docx]

**SUPPLEMENTARY MATERIALS**

**Topoisomerase I Activity and Camptothecin Sensitivity of Breast Cancer Derived Cell lines: A Comparative Study**

Cinzia Tesauro^1^, Anne Katrine Simonsen^1^, Marie Andersen^1^, Kamilla Wandsoe^1^, Emil Kristoffersen^1^, Line Algreen^1^, Noriko Hansen^1^, Anne Andersen^1^, Ann Katrine Jakobsen^2^, Magnus Stougaard^2^, Pavel Gromov^3^ and Birgitta R. Knudsen^1^*, Irina Gromova^3^*

^1^Department of Molecular Biology and Genetics, Aarhus University, Aarhus, Denmark

^2^Department of Pathology, Aarhus University Hospital, Aarhus Denmark

^3^ Danish Cancer Society Research Center, Genome Integrity Unit, Breast Cancer Biology Group, Copenhagen, Denmark

* to whom correspondence should be addressed: IG ([iig@cancer.dk](mailto:iig@cancer.dk)) and BRK ([brk@mbg.au.dk)](mailto:brk@mbg.au.dk))


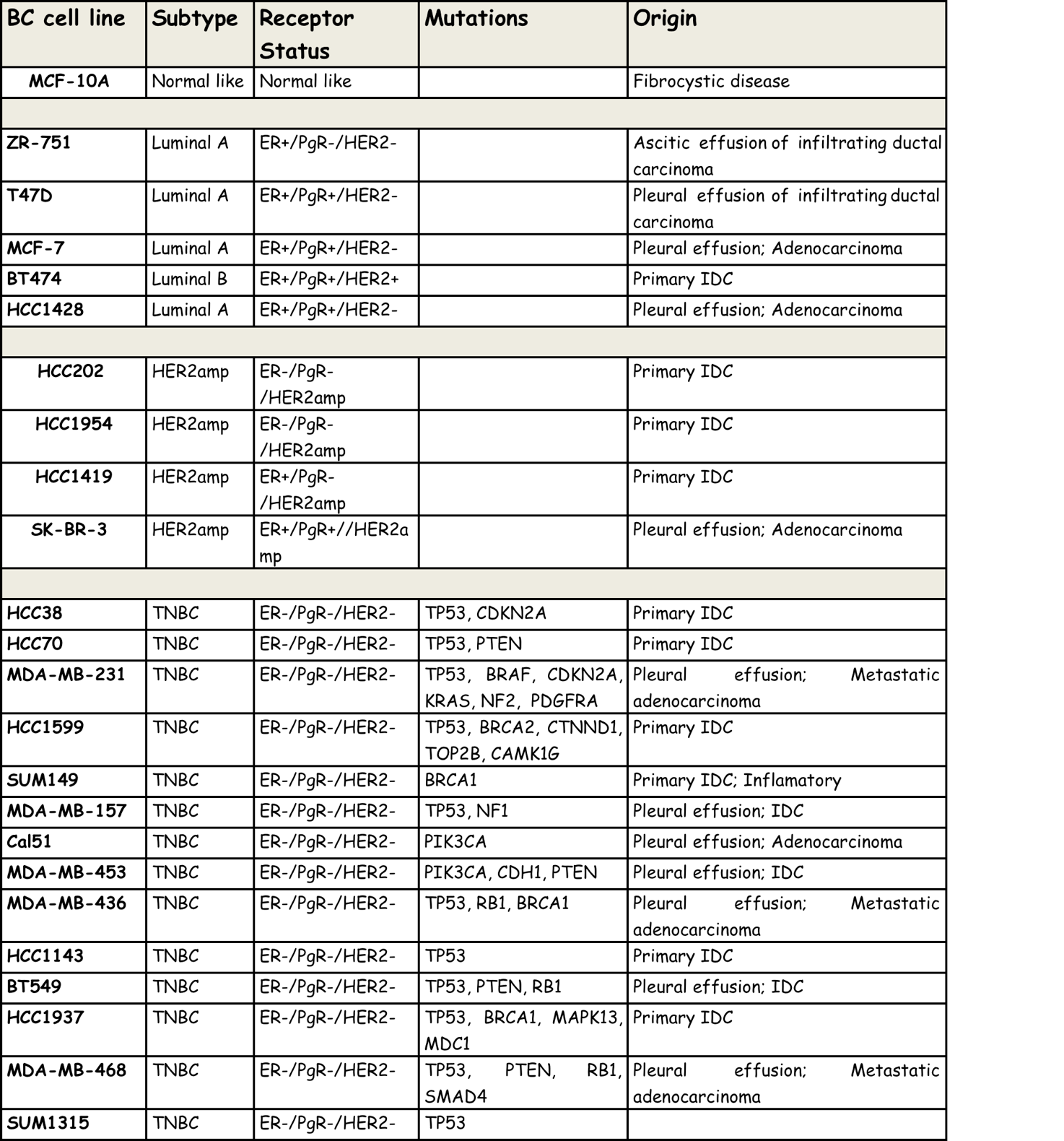


**Table S1:** Description of the cell lines used in the study. The corresponding information is from ATCC.

**Table S2:** Quantifications of the TOP1 protein level in cell extracts prepared from each cell line. TOP1 band intensities were normalized to the signal intensity of the actin detected on the same blot.


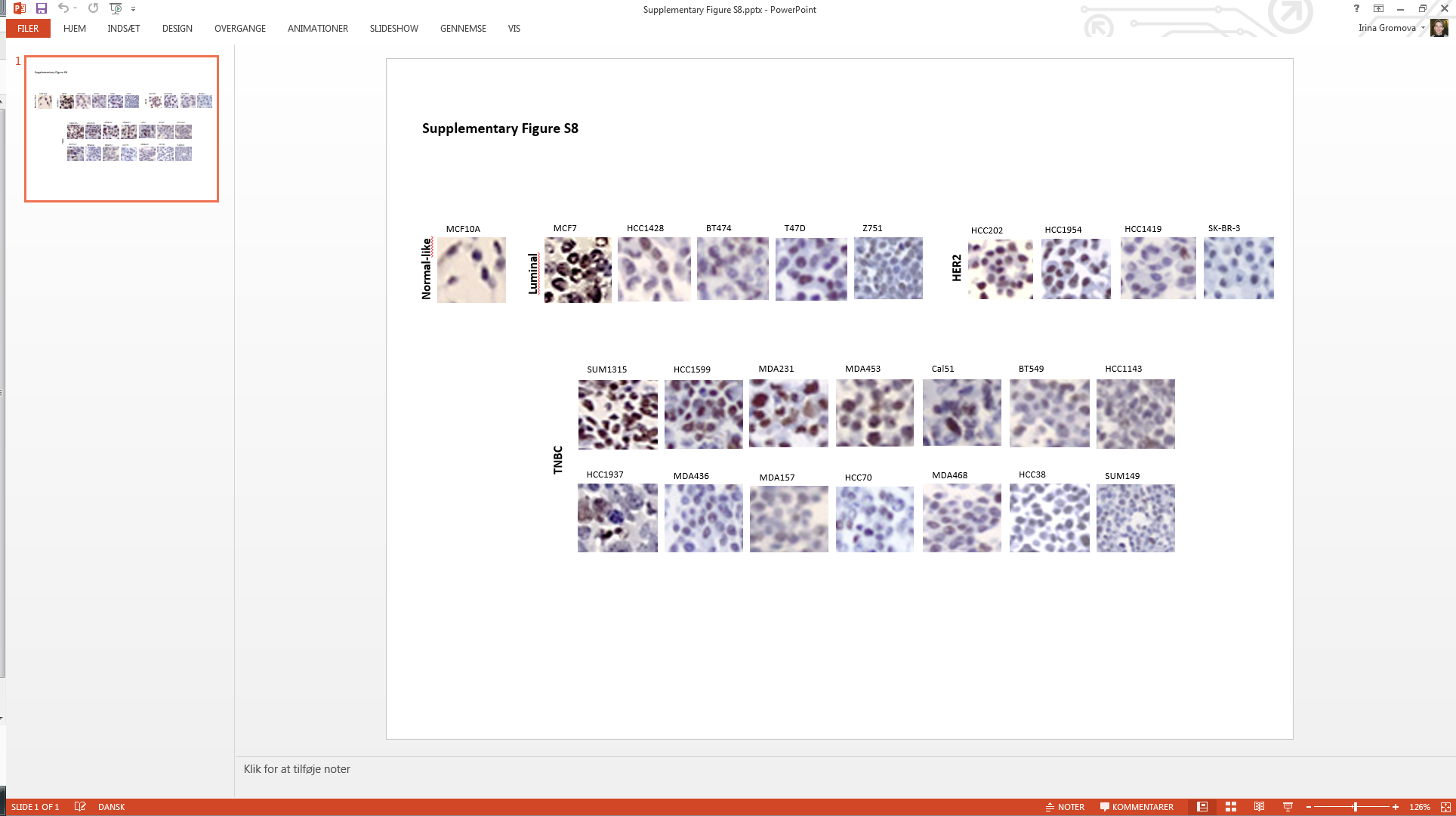


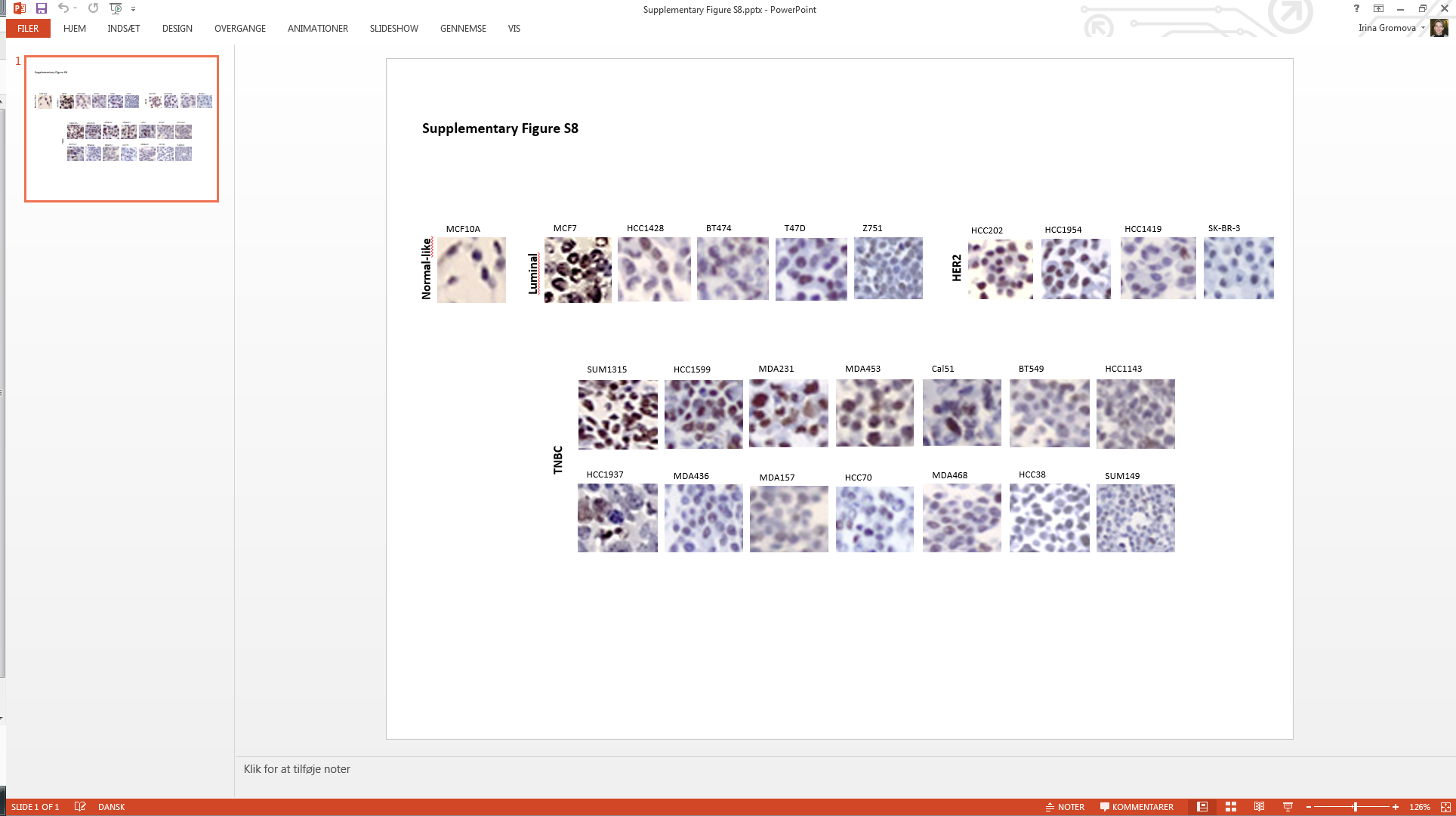


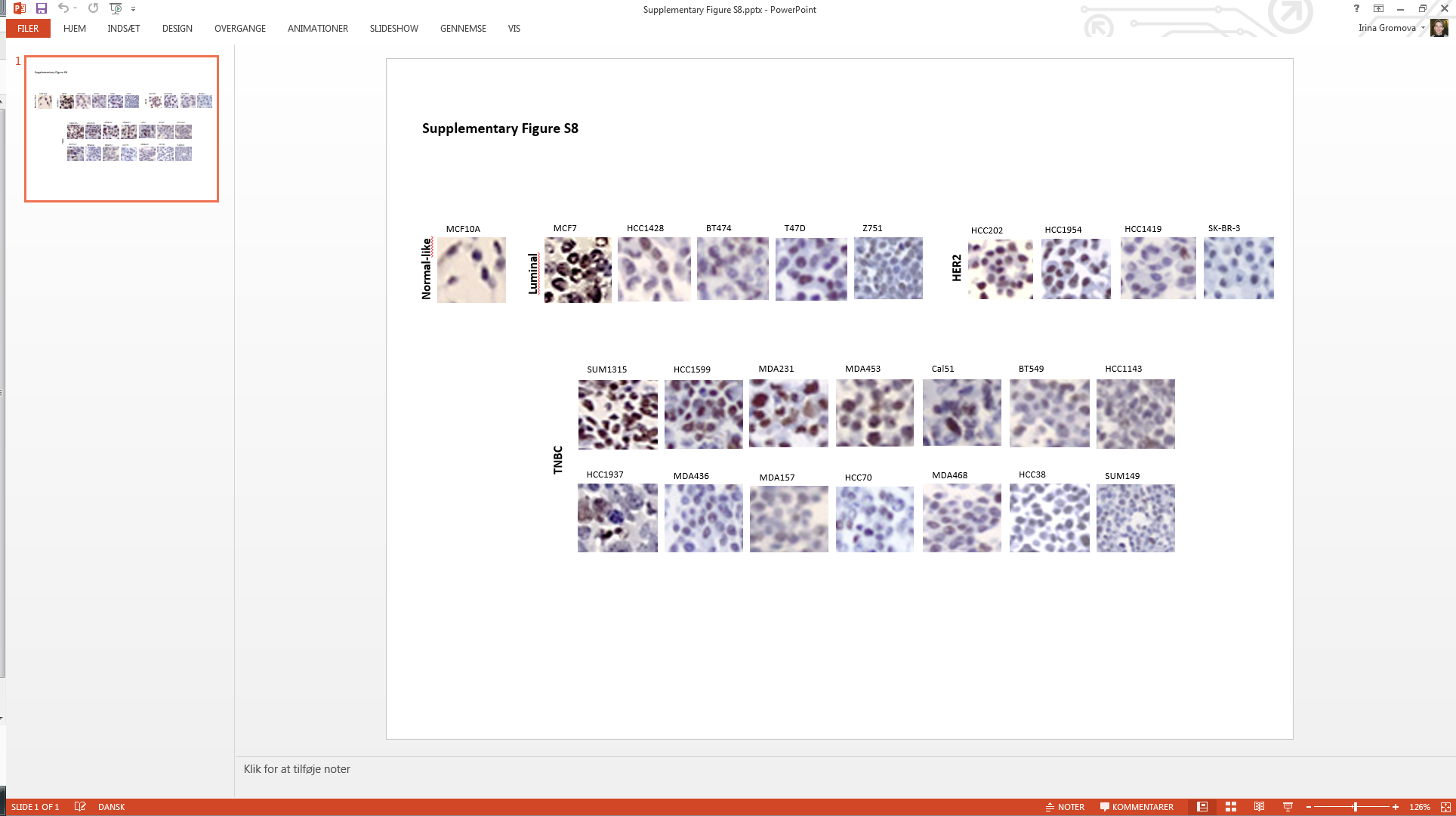


**Figure S1.** The magnified images (x40) of nuclear TOP1 IHC staining of all BC cell lines analyzed.


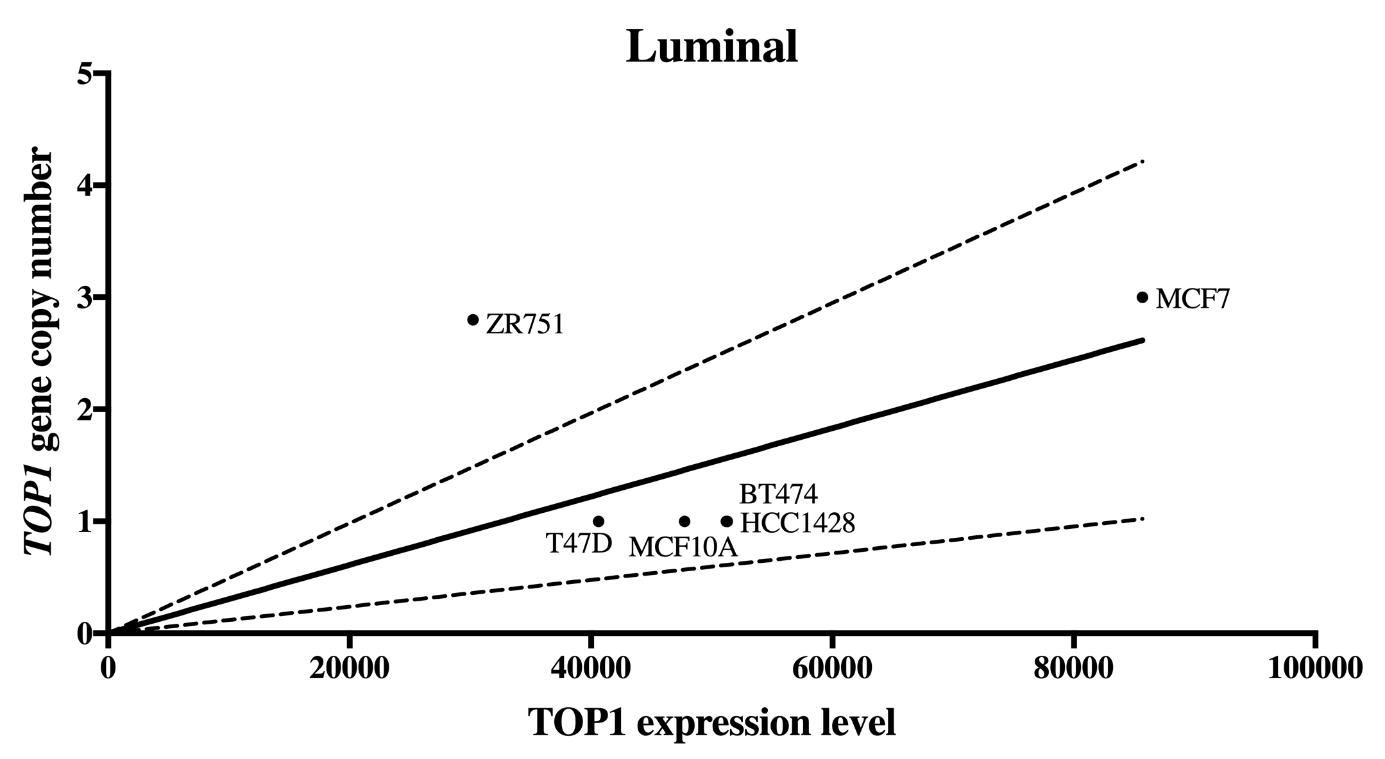


| ***TOP1* gene copy number vs**  **TOP1 expression level** |  |
| --- | --- |
| R squared | 0,1169 |
|  |  |
| P value (one-tailed) | 0,2535 |

**Figure S2.** Analysis of the correlation between the TOP1 protein expression level and the *TOP1* gene copy number for the cells lines of the luminal subtype. The table shows the R squared and p value estimated using the Spearman’s correlation method using Graph-pad prism software. Significance P<0.05.


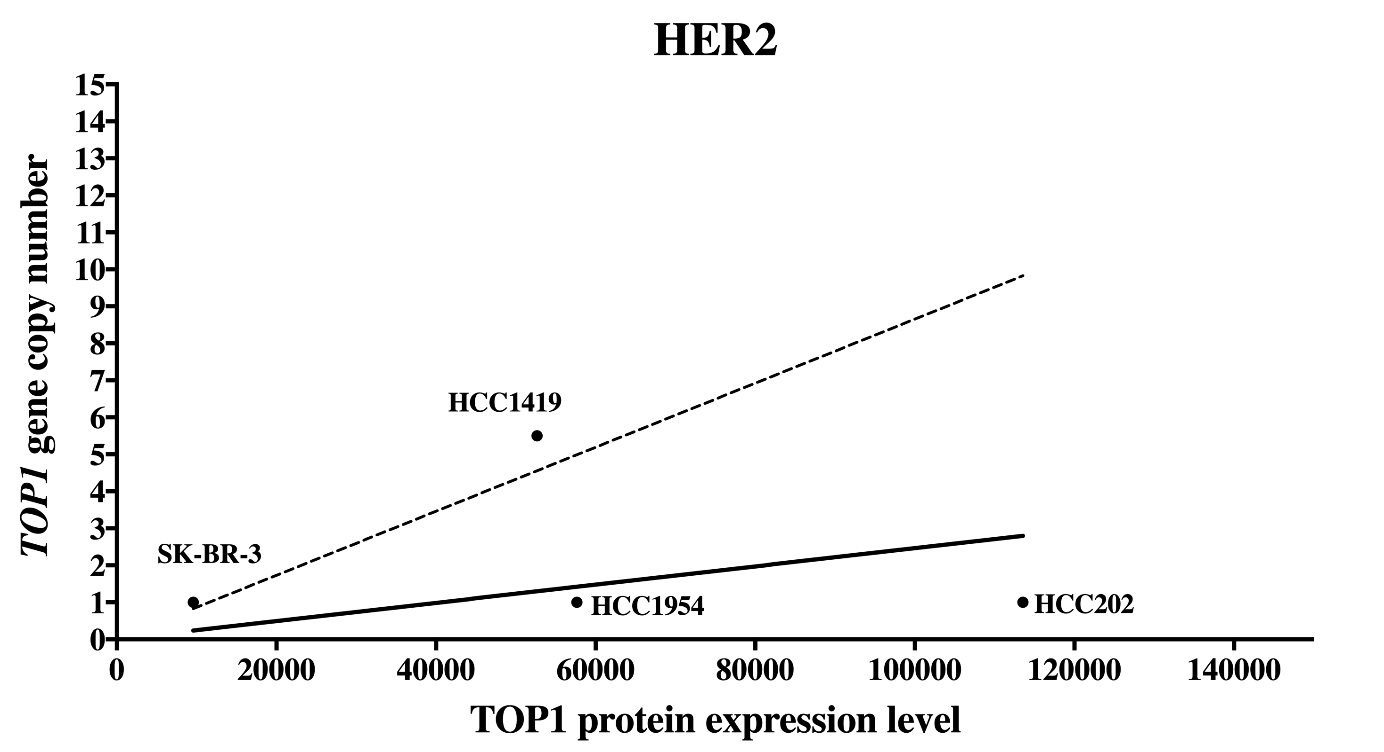


| ***TOP1* gene copy number vs**  **TOP1 expression level** |  |
| --- | --- |
| R squared | 0,007914 |
|  |  |
| P value (one-tailed) | 0,4555 |

**Figure S3.** Analysis of the correlation between the TOP1 protein expression level and the *TOP1* gene copy number for the cells lines of the HER2 subtype. The table shows the R squared and p value estimated using the Spearman’s correlation method using Graph-pad prism software. Significance P<0.05.


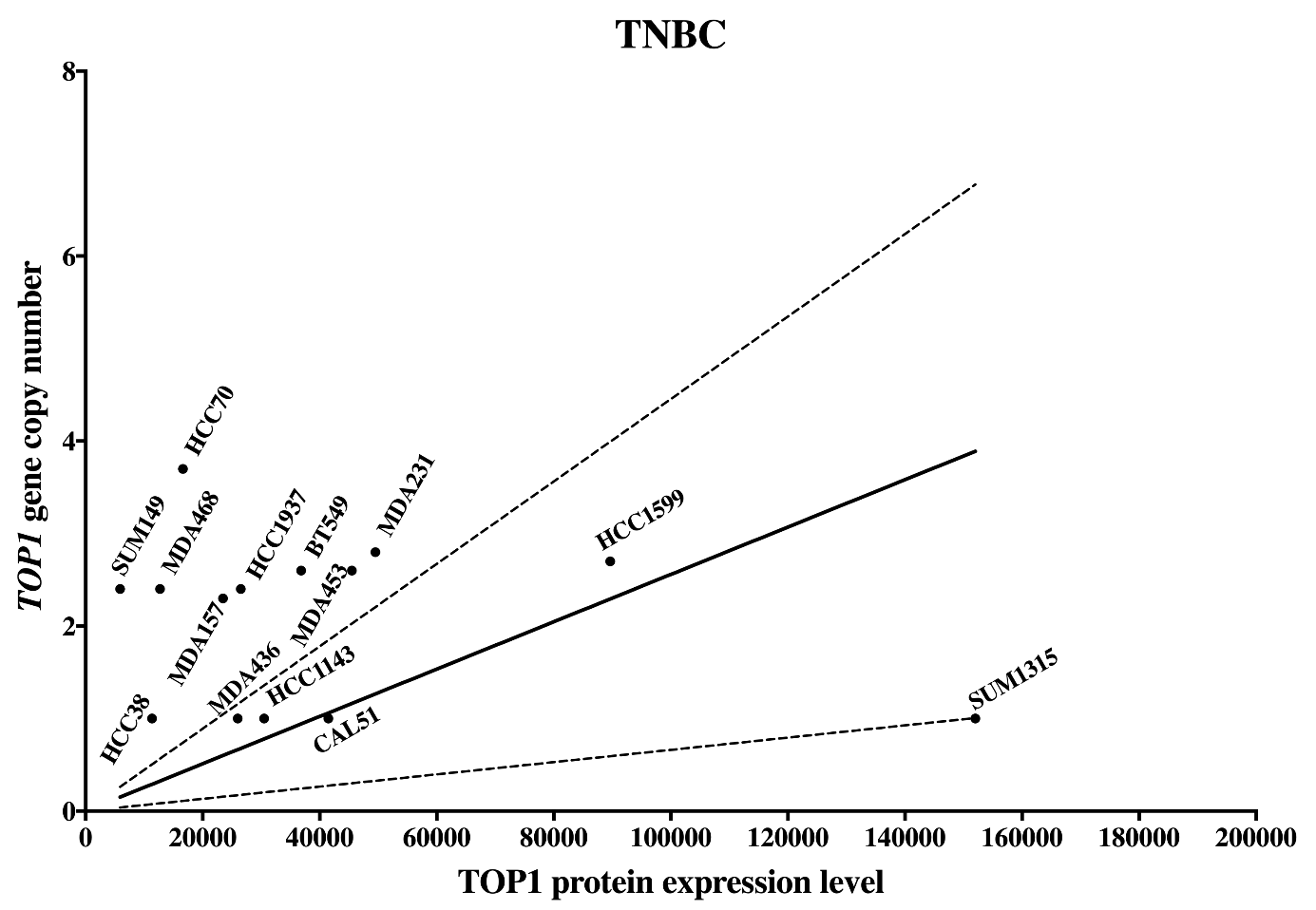


| ***TOP1* gene copy number vs**  **TOP1 expression level** |  |
| --- | --- |
| R squared | 0,04378 |
|  |  |
| P value (one-tailed) | 0,2364 |

**Figure S4**. Analysis of the correlation between the TOP1 protein expression level and the *TOP1* gene copy number for the cells lines of the TNBC subtype. The table shows the R squared and p value estimated using the Spearman’s correlation method using Graph-pad prism software. Significance P<0.05.


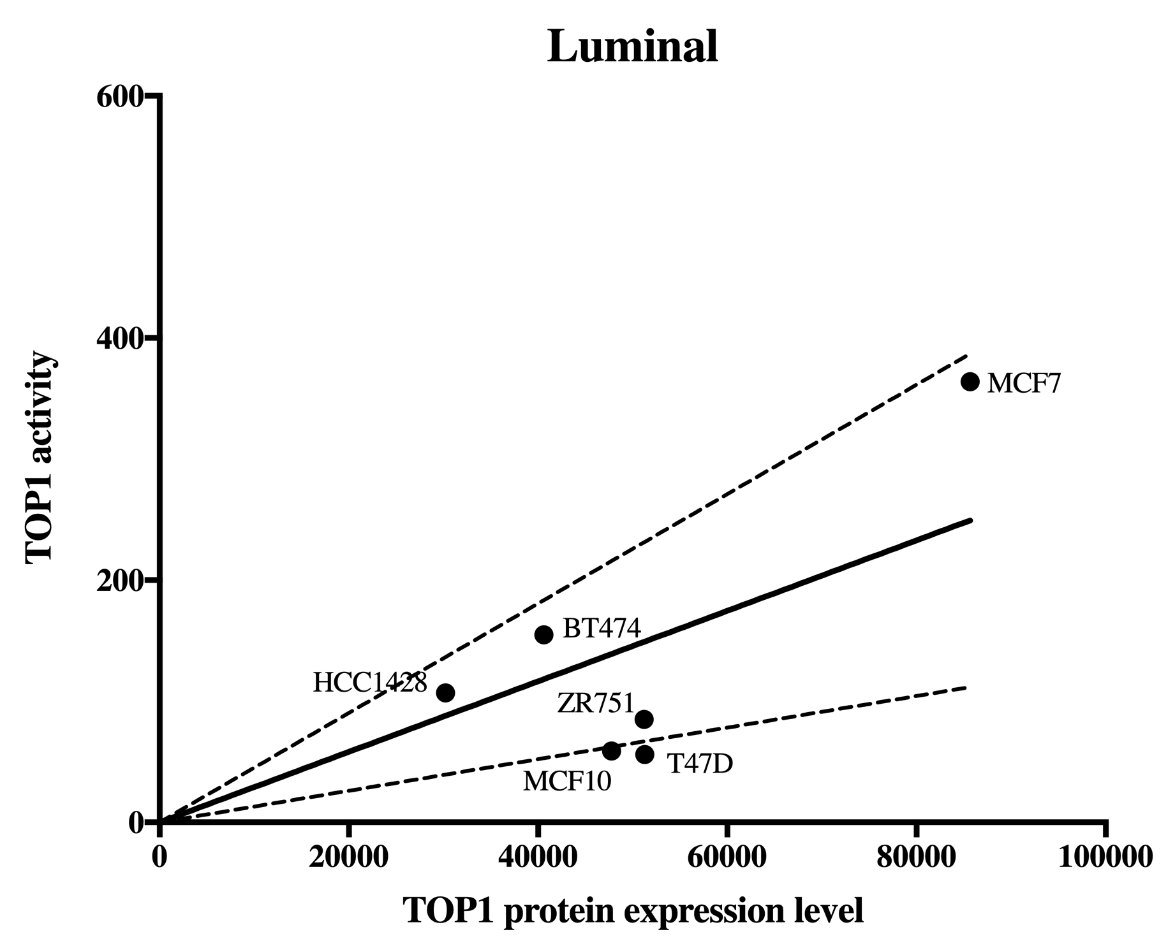


| **TOP1 activity**  **vs**  **TOP1 expression level** |  |
| --- | --- |
| R squared | 0,6093 |
|  |  |
| P value (one-tailed) | 0,0335 |

**Figure S5.** Analysis of the correlation between the TOP1 activity and the protein expression level for the cells lines of the luminal subtype. The table shows the R squared and P value estimated using the Spearman’s correlation method using Graph-pad prism software. Significance P<0.05.


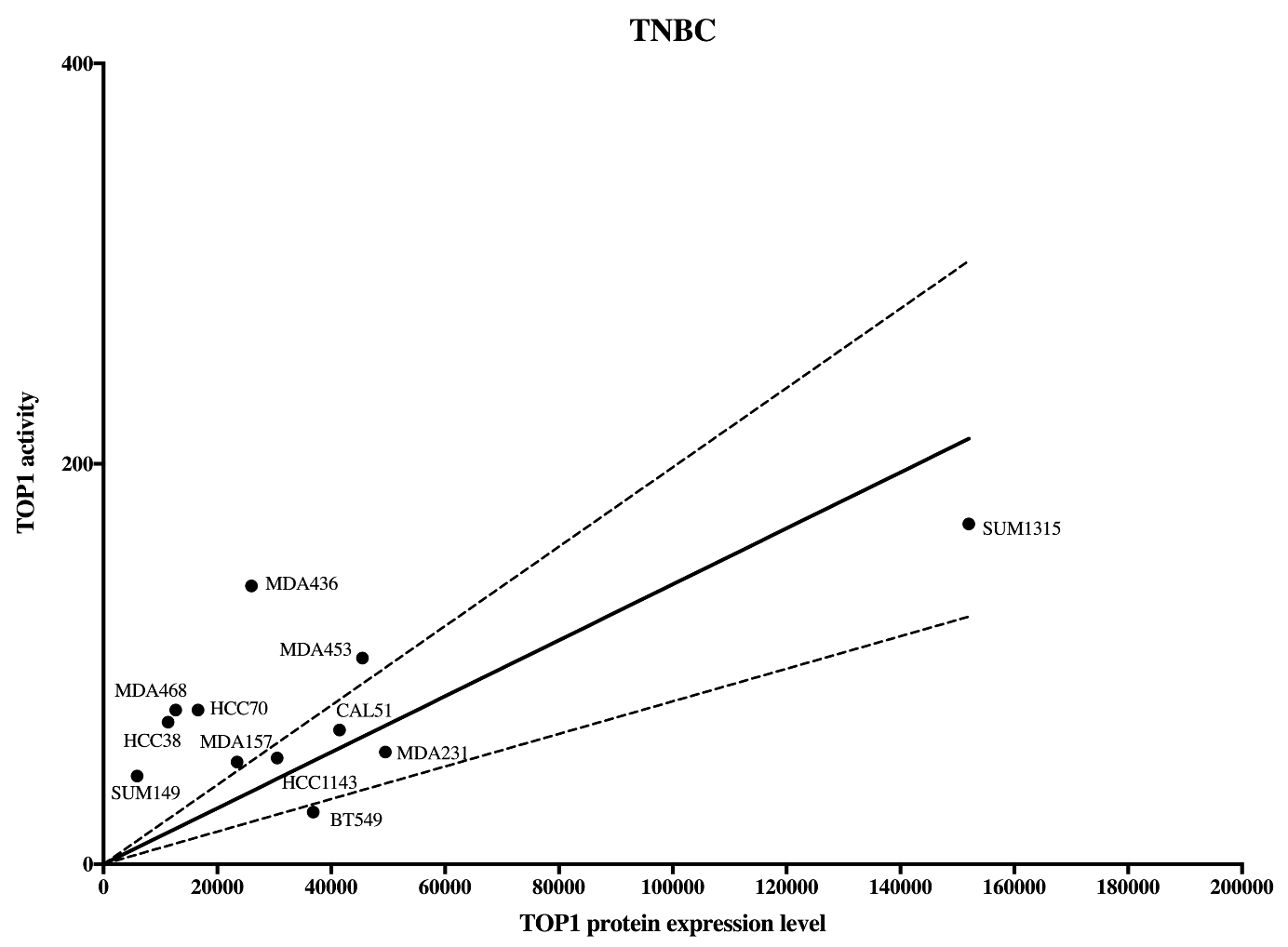


| **TOP1 activity**  **vs**  **TOP1 expression level** |  |
| --- | --- |
| R squared | 0,4392 |
|  |  |
| P value (one-tailed) | 0,0094 |

**Figure S6.** Analysis of the correlation between the TOP1 activity and the protein expression level for the cells lines of the TNBC subtype. The table shows the R squared and p value estimated using the Spearman’s correlation method using Graph-pad prism software. Significance P<0.05.


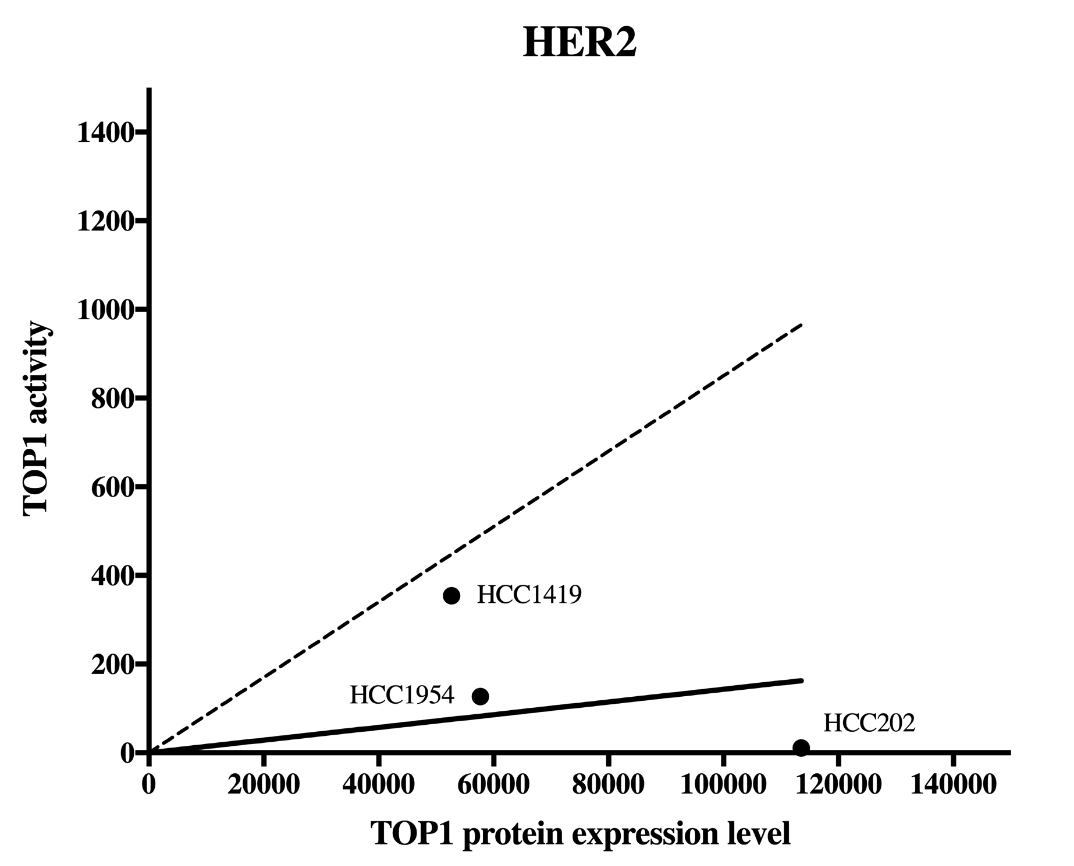


| **TOP1 activity**  **vs**  **TOP1 expression level** |  |
| --- | --- |
| R squared | 0,6488 |
|  |  |
| P value (one-tailed) | 0,2019 |

**Figure S7.** Analysis of the correlation between the TOP1 activity and the protein expression level for the cells lines of the HER2 subtype. The table shows the R squared and p value estimated using the Spearman’s correlation method using Graph-pad prism software. Significance P<0.05.


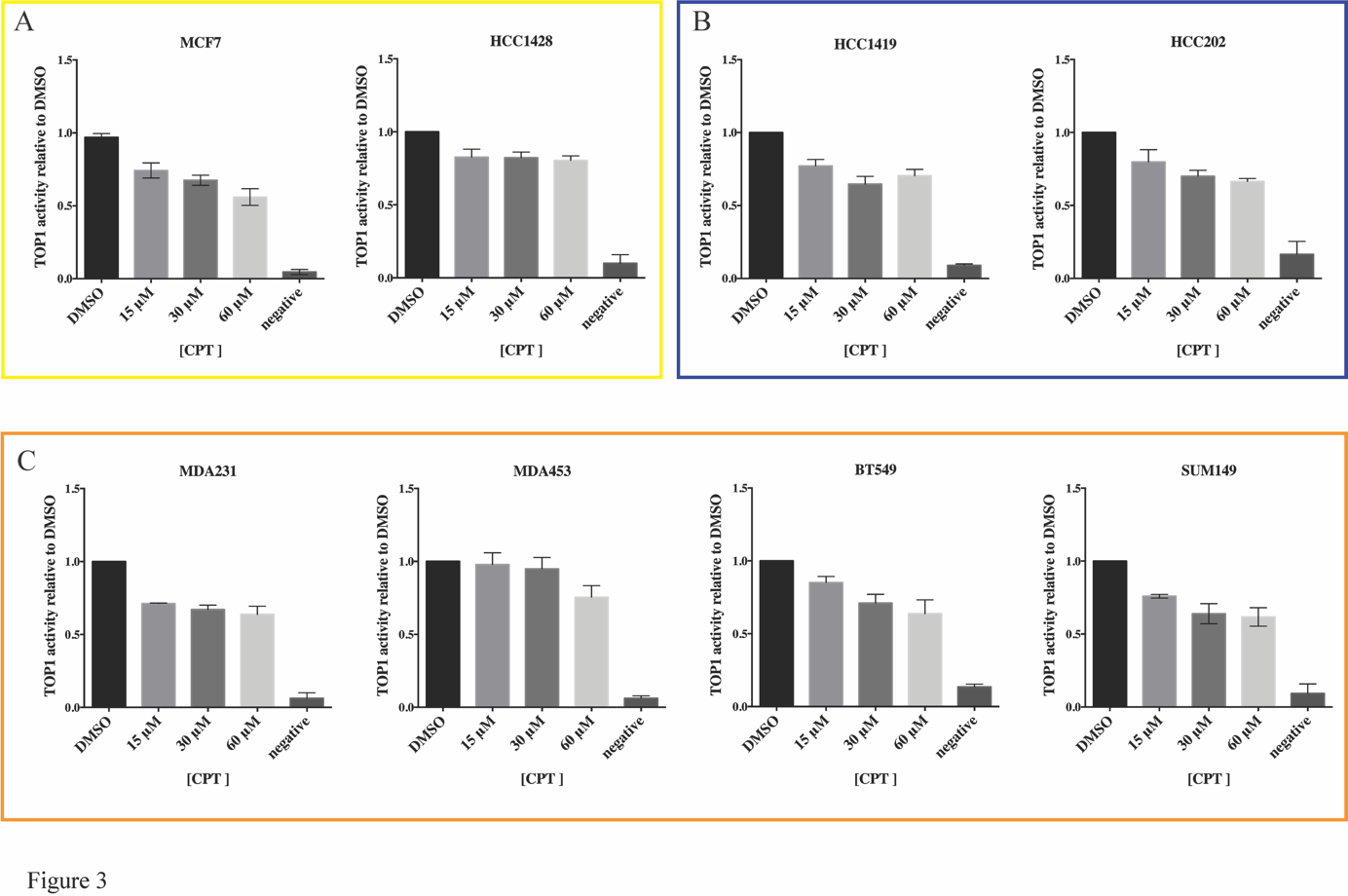


**Figure S8**. TOP1 susceptibility to CPT in nuclear extracts of the selected cell lines. TOP1 activity was measured in nuclear extracts from the selected cell lines as indicated in the figure after incubation with DMSO or increased concentrations of CPT from 15 to 60 μM using the REEAD assay. The intensities of REEAD signals obtained in the presence of CPT were normalized to the DMSO control for each cell line. Results were plotted as mean ± SD (four independent experiments). Yellow frames; cells lines of the luminal subtype, blue frames; cell lines of the HER2 positive subtype, orange frames cell lines of TNBC subtype.

| **Cell line** | **BC subtype** | **TOP1 gene copy number** | **TOP1 protein level** | **TOP1 enzymatic activity** | **Cell viability IC50 (μM)** | **TOP1 CPT susceptibility** | **TDP1 activity** | **Doubling time (hours)** |
| --- | --- | --- | --- | --- | --- | --- | --- | --- |
| MCF7 | Luminal | 3 | High | High | 0.089±0.017 | ns | ns | 33.08 |
| HCC1428 | Luminal | No amplification | Same as normal-like | Low | 0.448±0.054 | ns | ns | 98.47 |
| HCC202 | HER 2 | No amplification | High | Low | 0.481±0.060 | ns | ns | 89.47 |
| HCC1419 | HER 2 | 5.5 | Same as normal-like | High | 0.067±0.010 | ns | ns | 33.19 |
| MDAB231 | TNBC | 2.8 | Same as normal-like | Low | 0.040±0.011 | ns | ns | 26 |
| MDAMB453 | TNBC | 2.6 | Same as normal-like | Low | 0.058±0.019 | ns | ns | 21.87 |
| BT549 | TNBC | 2.6 | Same as normal-like | Low | 0.056±0.007 | ns | ns | 25.52 |
| SUM149 | TNBC | 2.4 | Low | Low | 0.065±0.010 | ns | ns | 26.2 |

**Table S3**: Summary of all the parameters investigated across the cell lines selected in the study. NS: Not significative differences between cell lines.
